# Fouling Analysis in One-Stage Ultrafiltration of Precipitation-Treated *Bacillus subtilis* Fermentation Liquors for Biosurfactant Recovery

**DOI:** 10.3390/membranes12111057

**Published:** 2022-10-28

**Authors:** Mai Lien Tran, Ying-Shr Chen, Ruey-Shin Juang

**Affiliations:** 1 Institute of Environmental Science, Engineering and Management, Industrial University of Ho Chi Minh City, Ho Chi Minh City 700000, Vietnam; 2 Department of Chemical and Materials Engineering, Chang Gung University, Guishan, Taoyuan 33302, Taiwan; 3 Department of Internal Medicine, Division of Nephrology, Chang Gung Memorial Hospital, Linkou, Taoyuan 33305, Taiwan; 4 Department of Safety, Health and Environmental Engineering, Ming Chi University of Technology, Taishan, New Taipei City 24301, Taiwan

**Keywords:** fouling analysis, one-stage ultrafiltration, primary recovery, surfactin, pretreated fermentation liquors

## Abstract

Primary recovery of surfactin from precipitation-pretreated fermentation broths of *Bacillus subtilis* ATCC 21332 culture by one-stage dead-end and cross-flow ultrafiltration (UF) was studied. Dead-end experiments were first performed to select suitable conditions, including the amount of added ethanol—a micelle-destabilizing solvent (0–70 vol%), type (polyethersulfone, polyacrylonitrile, poly(vinylidene fluoride)) and molecular-weight cut-off (MWCO, 30–100 kDa) of the membrane in the surfactin concentration range of 0.25–1.23 g/L. Then, the cross-flow UF experiments were conducted to check the recovery performance in the ranges of feed surfactin concentration of 1.13–2.67 g/L, flow velocity of 0.025–0.05 m/s, and transmembrane pressure of 40–100 kPa. The Hermia model was also used to clarify membrane fouling mechanisms. Finally, three cleaning agents and two in situ cleaning ways (flush and back-flush) were selected to regain the permeate flux. As for the primary recovery of surfactin from the permeate in cross-flow UF, a polyethersulfone membrane with 100-kDa MWCO was suggested, and the NaOH solution at pH 11 was used for membrane flushing.

## 1. Introduction

Currently, surfactants are exclusively synthesized from fossil oils. Much interest has recently been attracted to surface active microbial products as an alternative source of surfactants. They are biosurfactants, which have some advantages such as low toxicity, high biodegradability and biocompatibility, and low critical micellar concentration (cmc), as well as diversity relative to chemically synthesized surfactants [1]. Therefore, they are suited for some environmental issues, such as the dispersion of oil spills and bioremediation [2]. Moreover, biosurfactants have been applied in the petroleum industries, such as enhanced oil recovery [3], as well as in the cosmetic, food, and healthcare industries [4]. Biosurfactants have also been found for their potential applications in water and wastewater treatment from various sources [5].

The potential of biochemical production mainly lies in the development of simpler processes and the use of cheaper resources, which explains 10–30% of the overall cost [6]. At present, more high-value biochemicals are produced using fermentation methods, causing new challenges to purification and recovery steps, which count for the economy of the process and the labile nature of most of the molecules. In many biochemical processes, downstream processing usually accounts for up to 60% of the overall cost [7,8]. Therefore, research that can result in a high enough productivity compatible with the economic needs of biosurfactants is very crucial.

Surfactin is a heptapeptide linked to a β-hydroxy fatty acid comprising 14 or 15 carbon atoms, which represents one of the most powerful biosurfactants produced by several strains of *Bacillus subtilis* [9,10]. Basically, surfactin has excellent surface-active power; for example, it can lower the surface tension of deionized water from 72 to 27 mN/m even at a low concentration of 20 μM. The common recovery methods for surfactin from fermentation broth include foam separation, acid precipitation, or the combined both [11]; however, they gave a low purity (<60%). Some alternatives, such as extraction with water-immiscible solvents, adsorption chromatography, and thin-layer chromatography, have been employed [8,12]. The latter three methods generally suffer from high cost coupled with the use of a large quantity of highly toxic solvents like dichloromethane and chloroform or the loss of surface activity of surfactin. Such expensive and environmentally unfriendly nature makes them less practical. It is, therefore, highly desired to develop a more environmentally-friendly and economical method to improve the recovery and/or purity.

Membrane processes have attracted the attention of chemical and biochemical engineers due to their nature of efficient separation and energy-saving in comparison with other unit operations [10]. The use of membrane technologies for bioseparations has become promising due to their capability for size- and/or charge-based protein separation with high throughput [13]. Ultrafiltration (UF) is a pressure-driven membrane separation process for dissolved and suspended species with different particle or molecular sizes. The features of UF, including the minimized damage of biological molecules from shear effects, minimal denaturation, the avoidance of re-solubilization behavior, and high recovery/throughput, make it excellent in many applications [10,14]. At a level above the cmc, the surfactant will associate to form supramolecular structures like micelles or vesicles, with a nominal diameter up to two to three orders of magnitude larger than that of a single unassociated molecule. In this regard, surfactin micelles can be retained by UF membrane with a sufficiently low molecular weight cut-off (MWCO).

Although there are many benefits to UF processes, some limitations have remained. One of the major obstacles is the flux decline over time due to membrane fouling, which could be a result of the deposition of particles on the membrane surface and/or the blocking inside membrane pores. Due to membrane fouling, the performance of UF processes, including permeate flux and solute rejection, could be affected [15]. Moreover, the membrane fouling mechanism may be changed with the types, materials, and properties of membranes, as well as the feed composition, pressure, and filtration mode. Therefore, it is important to clarify the mechanisms of membrane fouling in a specific UF process for effective separation.

The aim of this study was to primarily recover surfactin from the fermentation liquors treated after acid precipitation and alkaline dissolution by one-stage dead-end and cross-flow UF. Higher recovery was the main target to first screen the experimental conditions, including the amount of micelle-destabilizing solvent ethanol, type, and MWCO of the membrane by dead-end UF. The mechanisms of membrane fouling were analyzed by the Hermia model and also via the so-called modified fouling index (MFI) [15,16]. Accordingly, flux decline and surfactin recovery were further tested using cross-flow UF. Moreover, the solution and the type for in situ membrane cleaning were selected.

## 2. Materials and Methods

### 2.1. Microorganisms and Culture Conditions

Surfactin was produced by *Bacillus subtilis* ATCC 21332 culture in this study. The nutrient broth (NB) medium contained 3 g/L of beef extract, 5 g/L of peptone, and mineral salt (MS) medium at pH 7. The MS medium contained 40 g/L of glucose, 50 mM of NH_4_NO_3_, 30 mM of KH_2_PO_4_, 40 mM of Na_2_HPO_4_, 7 μM of CaCl_2_, 0.8 mM of MgSO_4_, 4 μM of FeSO_4_, and 4 μM of tetrasodium salt of EDTA [9,17]. The pH was adjusted to 7 by adding 0.1 M of NaOH or HCl. Before use, the deionized water, produced by the Millipore Milli-Q system, and the MS medium were sterilized in an autoclave at 121 °C for 15 min. All analytical-reagent-grade inorganic chemicals were offered by Merck Co.

*B. subtilis* culture was taken from −80 °C frozen stock and was transferred onto agar medium for pre-culture. The culture (1 mL) was inoculated into a 250-mL flask containing 100 mL of NB medium at 30 °C and agitated at 200 rpm. After growing up to the late exponential phase (near 14 h), the NB medium was inoculated and fermented in a 5-L fermenter with a working volume of 4 L for another 4 days at 25 °C and 200 rpm.

The fermentation liquor was centrifuged at 10,000× *g* to remove the possible impurities, and the supernatant was then precipitated by adding 1 M of HCl until its pH being 4. The yellowish crude powder was obtained by centrifugation at 10,000× *g* for 15 min and drying in an oven at 37 °C for 2 days. After that, the powder was dissolved in NaOH solution (pH 11), and the resulting solution was used as the feed of UF experiments. The crude powder had a surfactin purity of approximately 55%, according to the method stated in Section 2.2.

### 2.2. Determination of Surfactin Concentration

After the culture samples were taken by centrifuge at 12,000× *g* for 15 min to remove the biomass, the concentration of surfactin in the supernatant was analyzed by reverse phase C18 HPLC at 30 °C, which is equipped with a Merck C18 column (5 μm) [18]. Prior to analysis, the samples were filtered through a Millipore microfilter (0.45 μm). A mixture of acetonitrile and 3.8 mM of trifluoroacetic acid (20 vol%) was used as the mobile phase, flowing at 1.0 mL/min. The sample (20 μL) was injected and analyzed at a wavelength of 205 nm using a UV detector (Jasco 975, Tokyo, Japan). Each analysis was duplicated under identical conditions, and the reproducibility was mostly within 5%.

Surfactin powder (98% purity as per label claim) directly obtained from Sigma Co. (St. Louis, MO, USA) was treated as the standard. The purity of surfactin in the sample taken in this study was determined by

(1) $$purity\left(\%\right)=\left(\frac{amount\;of\;surfactin\;determined\;by\;HPLC}{weight\;of\;dried\;sample\;powder\;dissolved\;in\;the\;solution}\right)\times 98\%$$

### 2.3. Apparatus, Membranes, and UF Experiments

**Figure 1:**
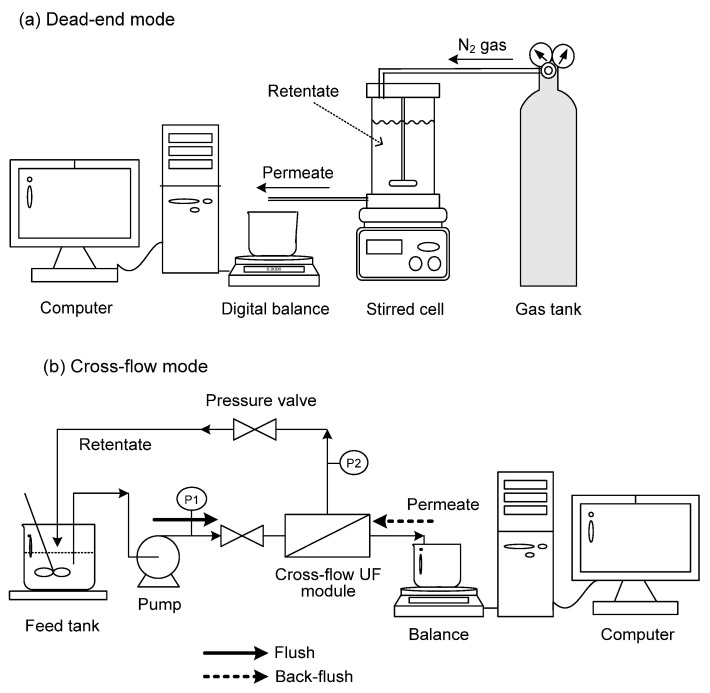
Schematic experimental setup for UF in (**a**) dead-end and (**b**) cross-flow modes.

UF experiments were conducted here in dead-end and cross-flow modes. In the former mode, a batch-stirred cell (Millipore, Burlington, MA, USA, XFUF07601) with a total volume of 300 mL was used. The disc membrane had a diameter of 76 mm. The membranes tested included polyethersulfone (PES, Millipore Co., Burlington, MA, USA), regenerated cellulose (YM, Millipore Co.), polyacrylonitrile (PAN, Osmonics Co., Bayan Lepas, Malaysia), and poly(vinylidene fluoride) (PVDF, Osmonics Co.). The nominal MWCOs of these membranes provided by the manufacturers were 30 kDa (YM 30, PVDF 30), 50 kDa (PES 50), and 100 kDa (PES 100, PAN 100). Figure 1a shows the experimental setup. The pressure was controlled by nitrogen gas. The UF cell was stirred at 300 rpm by a magnetic motor, which was far enough from the membrane, such that it could prevent the formation of a serious vortex within the cell. Preliminary tests had shown that the flux and surfactin rejection were little affected when the stirring speed exceeded 300 rpm.

In cross-flow mode, a flat-plate module (Millipore Minitan^TM^ system) was used, where the membrane had a length of 105 mm and a width of 62 mm with a filtration area of 65.1 cm^2^. This system (Figure 1b) was equipped with pressure gauges in the inlet and outlet of the retentate. The liquor was fed to the module using a Masterflex peristaltic pump (Model 7518-10). The membrane was mounted in a chamber with two acrylic panels, and UF experiments were performed at 25 °C with a volume of 500 mL. The cross-flow velocity and transmembrane pressure (TMP) were adjusted by the valve and pump controller, where TMP was calculated by

(2) $$\mathrm{TMP}=\left(\frac{P_{in}+P_{out}}{2}\right)-P_{permeate}$$

where *P_permeate_*, *P_in_*, and *P_out_* are the pressures in the permeate side, feed side, and retentate side, respectively (kPa).

Because of the varying composition of the retentate and the permeate with filtration time in both UF modes, the rejection of surfactin (*R*) at a pseudo-steady state was calculated by

(3) $$R\left(\%\right)=\left[1-\left(\frac{C_{p}}{C_{0}}\right)\right]\times 100\%$$

where *C*_0_ and *C_p_* are the concentrations of surfactin in the feed and permeate (g/L), respectively. The recovery of surfactin when 60% of the working volume being permeated was calculated by:

(4) $$recovery\left(\%\right)=\left(\frac{C_{p}}{C_{0}}\right)\times 100\%$$

In the present system, the *recovery* (%) was almost equal to (100% − *R*) when surfactin product could pass through the membrane.

### 2.4. Determination of Modified Fouling Index (MFI)

**Figure 2:**
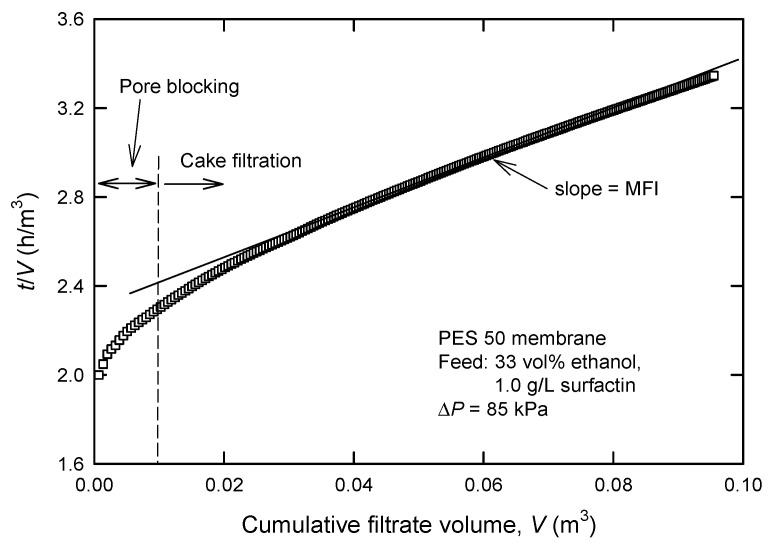
Typical plot for the determination of modified fouling index (MFI) in UF processes.

Because the tested membranes yield different pure water fluxes even at a fixed applied pressure (Δ*P*), a more rigorous way is required to screen the suitable membrane. The typical membrane filtration generally reveals three consecutive regions; that is, pore blocking, cake filtration, and cake filtration with compression [16]. Firstly, deposition of the particles blocking the entrance to the pore or inside the membrane pores will cause a sharp rise in slope, as shown in Figure 2.

The typical curve is then followed by a minimum linear slope in the second region, where particles deposit on the surface of the membrane. The so-called modified fouling index (MFI) is essentially based on cake filtration (region 2); in this case, particles are retained on the membrane surface as a cake. The cake will add an additional resistance (*R_c_*) to the resistance of the membrane itself (*R_m_*); therefore, flux decline under constant pressure filtration can be described by [15,18]:

(5) $$\frac{1}{A}\left(\frac{dV}{dt}\right)=\frac{\Delta P}{\eta \left(R_{m}+R_{c}\right)}$$

where *V* is the cumulative volume of the permeate (m^3^), *t* is the time (h), Δ*P* is the applied pressure (Pa), *η* is the viscosity of the permeate (Pa s), and *A* is the membrane area (m^2^). If there is no cake compression (this will be checked later), then

(6) $$R_{c}=\left(\frac{V}{A}\right)I$$

where *I* denote a measure of the fouling potential of the permeate (m^−2^).

Finally, we obtain the following equation by combining Equations (5) and (6) and integrating it at constant pressure

(7) $$\frac{t}{V}=\frac{\eta \cdot R_{m}}{A\cdot \Delta P}+\left(\frac{\eta \cdot I}{2A^{2}\Delta P}\right)V$$

The MFI is defined as the slope of the linear region in the plot of (*t*/*V*) vs. *V* (Figure 2).

### 2.5. Cleaning of the Used Membranes

The membrane was in situ cleaned during cross-flow UF for 15 min in the same direction as the filtration runs (flush) or in the reverse direction (back-flush), as shown in Figure 1b. Deionized water, NaOH solution (pH 11), and 1 wt.% Terg-A-zyme solution (Alconox, Inc., White Plains, NY, USA) were tested as a cleaning solution. Here, cleaning experiments were conducted at 25 °C with an initial feed volume of 800 mL.

When cross-flow UF experiments were completed, the used membranes were flushed immediately with NaOH solution (pH 13), 1 wt.% Terg-A-zyme solution, and deionized water in sequence for 30 min to recover the permeability. They were finally stored in 0.1 M of NaOH solution overnight at 4 °C. The cleaned membrane was repeatedly employed only if the difference in pure water flux between the cleaned and pristine membranes was smaller than 5%.

## 3. Results and Discussion

### 3.1. Flux in Dead-End UF and Surfactin Recovery

It has been reported that the raw culture liquor with *B. subtilis* ATCC 21332 consists of many molecules with different molecular weights (Table 1) [19]. Owing to the presence of surfactin (both forms of monomer and micelle) and several small molecules, the pressure-driven membrane separation process based on size sieving effect such as UF is suggested. Figure 2 shows the fluxes and surfactin rejections by dead-end UF using various membranes in the presence of 33-vol% ethanol. It is known that organic solvents such as alcohols can destabilize surfactant micelles because these solvents could form a palisade-like structure through the interface of surfactant molecules [20]. The presence of ethanol will change the surface tension, resulting in affecting the wetting properties of the aqueous surfactant solution [21,22]. As reported by Isa et al. [23], surfactin monomer will be collected in the permeate, while protein macromolecules are retained by the membrane. Here, ethanol was selected as the surfactin micelle-destabilized reagent for this purpose.

**Figure 3:**
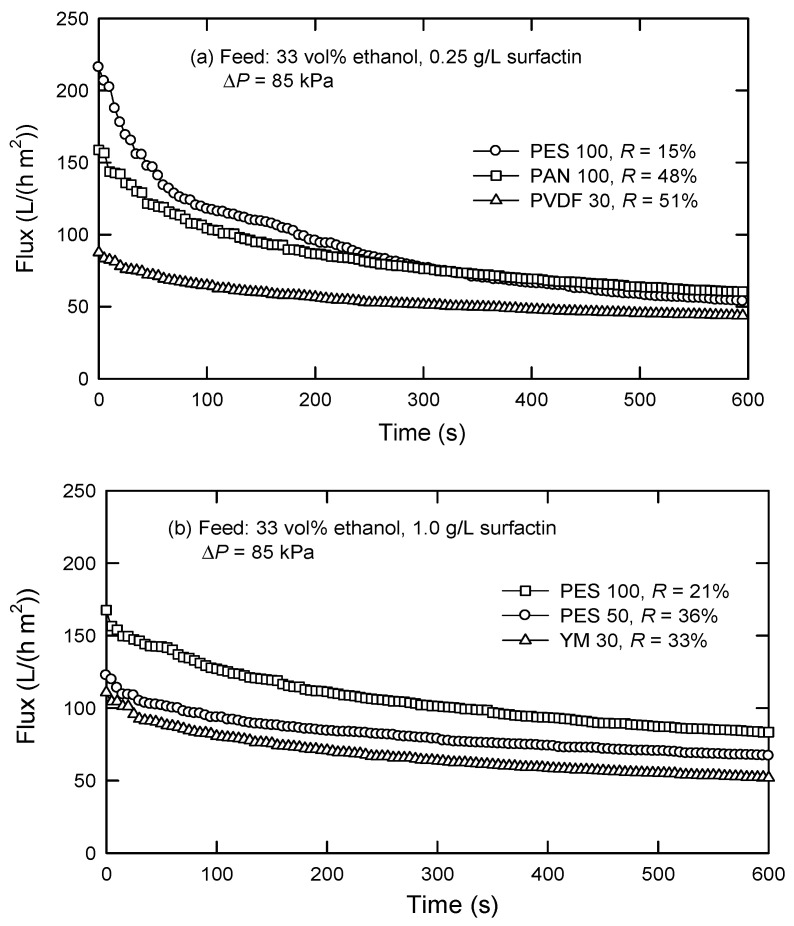
Flux decline and surfactin rejection in the presence of 33 vol% ethanol and a feed surfactin concentration of (**a**) 0.25 g/L and (**b**)1.0 g/L by dead-end UF.

It is evident that surfactin rejection changes with the types of membranes. As shown in Figure 3, the membrane with lower MWCO reveals a higher surfactin rejection. For instance, PES 100 presents an *R*-value of 21%, which increases to 36% in the case of PES 50 (Figure 2b). This is similar to the case of PES 100 and PVDF 30, with rejections of 15% and 51%, respectively. For the membranes with the same MWCO, such as PES 100 and PAN 100, the rejection notably varies. This could be a result of different membrane hydrophilicities; in this case, water contact angles of PES, PAN, PVDF, and YM membranes were measured to be 62.9°, 58.4°, 129°, and 15.7°, respectively. PAN 100 membrane with a lower contact angle than PES 100 membrane reveals a higher permeability, resulting in a lower *C*_p_ and a higher surfactin rejection (*R*). The results deduced that surfactin could be separated from other protein macromolecules by UF after the micelles are dissociated to monomers. Among the membranes studied, PES 100 has the lowest surfactin rejection but gives a comparatively high flux up to 51–73 L/(h m^2^).

Table 2 lists the MFI values calculated according to Equation (7), showing that MFI increases with a decreased MWCO for a specific membrane material. These data support that PES 100 was suitable for further use. It is noted that cake filtration with compression (region 3) is not observed under the conditions studied.

**Figure 4:**
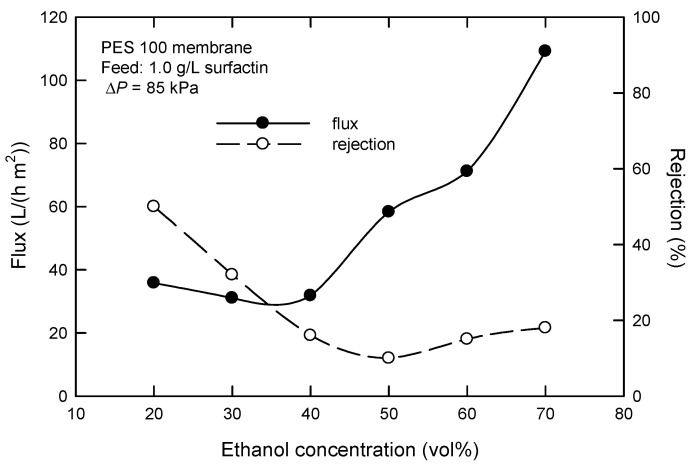
Effect of ethanol concentration on the flux and surfactin rejection in dead-end UF using PES 100 membrane.

The influence of the added amount of ethanol on the “steady” flux and surfactin rejection with the PES 100 membrane is shown in Figure 4. Generally speaking, flux increases, and surfactin rejection decreases as the amount of ethanol increases. This can be understood that increasing the ethanol amount implies a decreased surface tension of the water/ethanol solution of surfactin [21] and, hence, micelle stability or molecular size. Although ethanol can be recovered from the mixture of surfactin and ethanol using a rotary evaporator under reduced pressures, an ethanol amount of 33% is selected here when both factors of recycling use and ethanol loss are simultaneously considered. It is expected that the relatively low flux can be enhanced when UF is operated in continuous modes (e.g., cross-flow).

**Figure 5:**
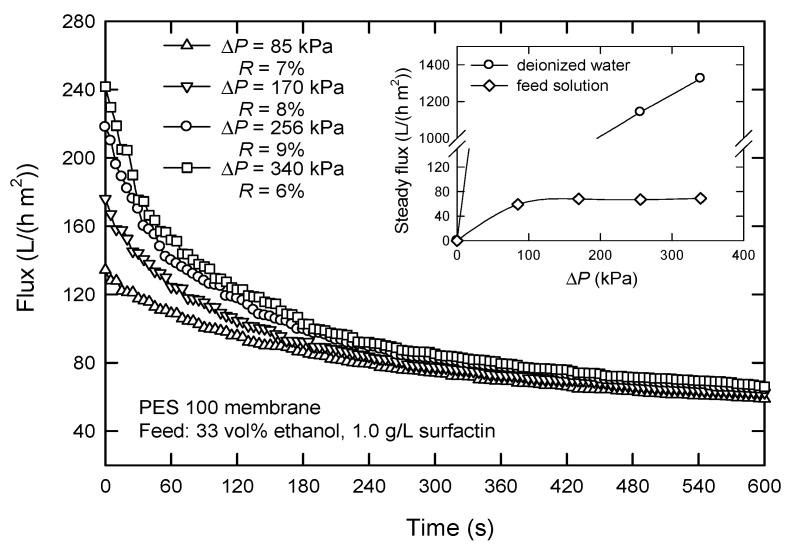
Effect of the applied pressure Δ*P* on the flux in dead-end UF using PES 100 membrane.

Figure 5 reveals the effect of applied pressure (Δ*P*) on the flux with the PES 100 membrane. It is seen that Δ*P* affects the flux at the beginning of a filtration process. The flux increases at a higher applied pressure. When the steady state reaches, the effect is negligibly small. At low Δ*P*, only a few molecules are deposited on the membrane surface, and thus, the steady flux is proportional to Δ*P* (see insert, Figure 5). When Δ*P* is increased, fouling occurs, and accumulation exists; in this case, flux no longer increases with Δ*P*. It appears that the flux is pressure independent when Δ*P* > 80 kPa, which shows a weak form of critical flux [24]. This type of critical flux is observed when some solutes are small enough to go into the membrane pores and are adsorbed onto the pore walls, which is favored by attractive electrostatic forces. Another reason is probably the accumulation of large molecules, such as polysaccharides, peptides, and proteins, on the membrane surface [25]. Thus, an applied pressure of 85 kPa was chosen in dead-end UF.

**Figure 6:**
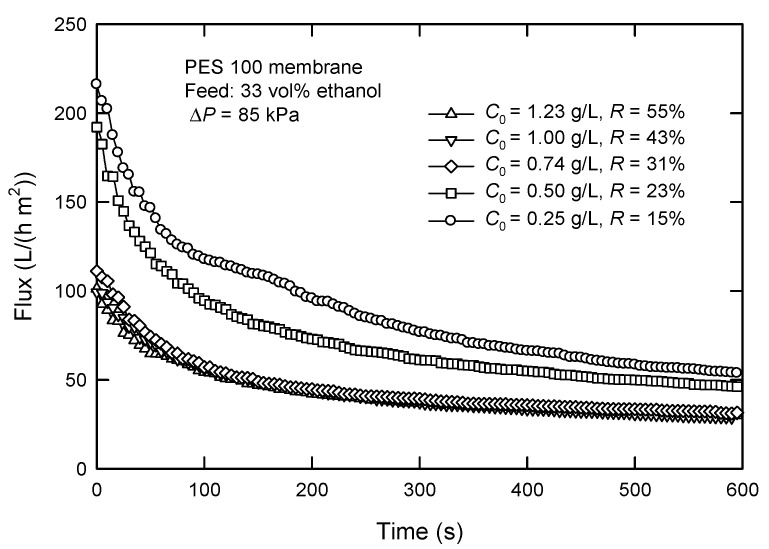
Effect of surfactin concentration *C*_0_ on the flux and surfactin rejection in dead-end UF.

Figure 6 shows the influence of surfactin concentration (*C*_0_) on the flux and surfactin rejection. The flux decreases with an increased *C*_0_. Particularly, the flux decreases from 53.9 to 46.2 L/(h m^2^) when *C*_0_ is 0.25 and 0.50 g/L, respectively, at a filtration time of 600 s. The flux reduces to 31.1 L/(h m^2^) and almost gets stable with the initial surfactin concentration higher than 0.74 g/L. This is likely caused by concentration polarization, which leads to a higher solute concentration close to the membrane surface than in bulk [26]. This will cause the further formation of the cake layer, particularly at sufficiently high concentrations, resulting in a significant increase in surfactin rejection and hence a lower surfactin recovery because the cake layer acts as a secondary membrane.

### 3.2. Flux in Cross-Flow UF and Surfactin Recovery

The screening results from dead-end UF tests, including the use of PES 100 membrane, and the addition of 33 vol% ethanol, were accordingly applied in cross-flow UF. In the cross-flow mode, the feed solution flows parallel to the membrane surface and permeates through the membrane, which can reduce the formation of the cake layer to keep it at a low level. On the other hand, PES has become one of the potential membrane materials because it has many merits, such as the availability of unlimited qualities, creep resistance, high rigidity, and good thermal stability [27].

**Figure 7:**
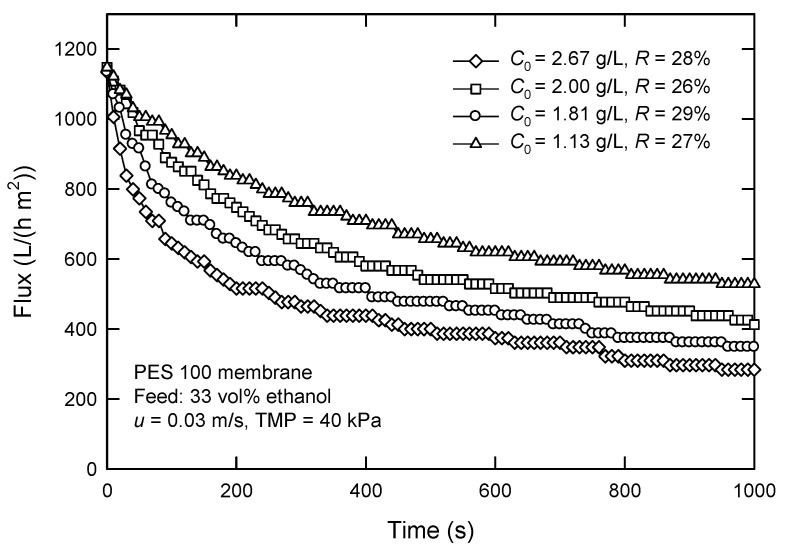
Effect of surfactin concentration *C*_0_ on the flux and surfactin rejection in cross-flow UF using PES 100 membrane.

The effect of *C*_0_ on the flux and surfactin rejection with the PES 100 membrane is shown in Figure 7. Apparently, the initial concentration significantly affects the flux. In particular, the flux decreases from 605 to 464 L/(h m^2^) when surfactin concentration increases from 1.13 to 2.67 g/L. In contrast to the dead-end mode, the flux is much improved under similar concentration ranges in cross-flow mode. The rejection of surfactin is 26–29%, much lower than that obtained in dead-end UF at corresponding *C*_0_ (>43%), as shown in Figure 6. This means that surfactin recovery from the permeate by cross-flow UF is more promising. Smaller molecules, including surfactin monomers, pass through the membrane, but most of the macromolecules are retained. It is noted that the recovered surfactin has a purity of about 75%. Furthermore, the steady flux is much higher than that obtained in dead-end UF, although the TMP in a cross-flow system is only 40 kPa.

**Figure 8:**
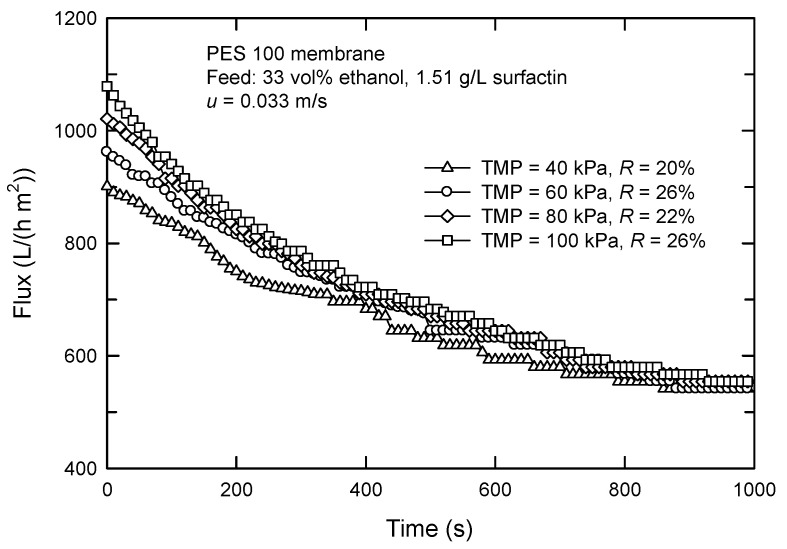
Effect of TMP on the flux in cross-flow UF using PES 100 membrane.

The performance for dead-end and cross-flow UF of various target solutions has been compared previously [28,29]. In the dead-end mode, the flow is perpendicular to the membrane surface, and the solution is pushed under applied pressure. This causes the accumulation of solutes as a layer on the membrane surface, leading to a reduction in flux. On the other hand, the feed stream in cross-flow mode is parallel to the membrane surface. The shear force reduces the accumulation of solutes and causes one to form a comparatively thinner cake. Actually, the selection of TMP is based on the experimental data, as shown in Figure 8. The higher TMP results in a faster decrease in flux. Moreover, a pseudo-steady state appears earlier at a lower TMP, which is probably due to the less effect of TMP on the filtration resistance of the membrane surface. The rejection was lowest in the case of TMP = 40 kPa; therefore, it was chosen for further experiments.

**Figure 9:**
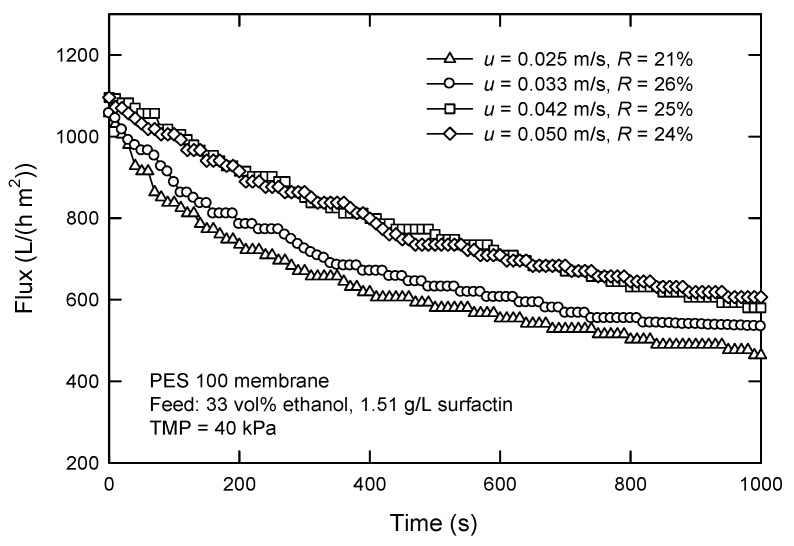
Effect of cross-flow velocity on the flux and surfactin rejection in cross-flow UF using PES 100 membrane.

Figure 9 shows the influence of cross-flow velocity (*u*) on the flux and surfactin rejection with the PES 100 membrane. Increasing *u* leads to an increased flux. The flux is just 464 L/(h m^2^) at a velocity of 0.025 m/s and rises to 605 L/(h m^2^) at 0.050 m/s. The rejection is in the range of 21–26%. The fluxes attenuate at the beginning of the filtration because of the formation of the cake layer. In fact, increasing *u* also results in an increased flux, an increased mass transfer, and a thinner boundary layer. This is because of the creation of turbulence and better hydrodynamic condition, where other particles have less opportunity to be deposited on the cake surface at higher *u*, leading to a thinner cake and a lower filtration resistance [30].

In summary, the recovery of surfactin from the permeate using “one-stage” dead-end and cross-flow modes was 55% and 75%, respectively, under the optimal conditions studied. Moreover, the purity of surfactin in the recovered products was approximately 75% using both UF modes. In contrast to our previous study by “two-stage” UF processes [31], most of the surfactin micelles were rejected by PES 100 membrane, and surfactin was further purified by PES 100 membrane after the surfactin micelles were dissociated by adding 33 vol% ethanol. This two-step route yielded the H-form surfactin with a purity of 85% and a recovery of 87% (feed concentration, 2.05 g/L). Our results indicated the application potential of the present “one-stage” UF process for surfactin recovery, although the purity and recovery of surfactin were slightly lower than those of the “two-stage” UF process.

### 3.3. Analysis of Membrane Fouling by the Hermia Model

To further predict the flux when using PES 100 membrane in cross-flow mode, the Hermia model is adopted [15]. Four types of fouling mechanisms, including complete blocking, intermediate blocking, standard blocking, and cake layer formation, are involved. Originally, the Hermia model was developed for dead-end mode; however, it was adapted for cross-flow configuration 16 and presented by Equation (8):

(8) $$-\frac{dJ}{dt}=K\left(J-J_{ss}\right)J^{2-n}$$

where *J* is the flux, *J*_ss_ is the pseudo-steady-state flux, *K* is a model constant, and *n* is the blocking index. Different *n* values describe different fouling mechanisms. When *n* = 2, it means complete blocking, stating that the entrance of membrane pores is fully blocked by solutes as a monolayer. The intermediate blocking one (*n* = 1) expresses that the solutes are deposited on the membrane surface without penetrating inside the pores; however, these molecules can form multiple layers. For the standard blocking model (*n* = 1.5), the size of the solute molecule is smaller than that of membrane pores; thus, these molecules go inside and deposit on the pore wall. The fourth model is cake layer formation (*n* = 0), presenting the accumulation of solutes whose size is much larger than that of membrane pores. The equation for each blocking mechanism is shown in Table 3. In each model, the fitness of linear plot ln *J* vs. *t* (*n* = 2), 1/*J* vs. *t* (*n* = 1), $1/\sqrt{J}$ vs. *t* (*n* = 1.5), or $1/\sqrt{J^{2}}$ vs. *t* (*n* = 0) was examined through the *R*^2^ value. The higher *R*^2^ value indicates a better fit of the model.

**Figure 10:**
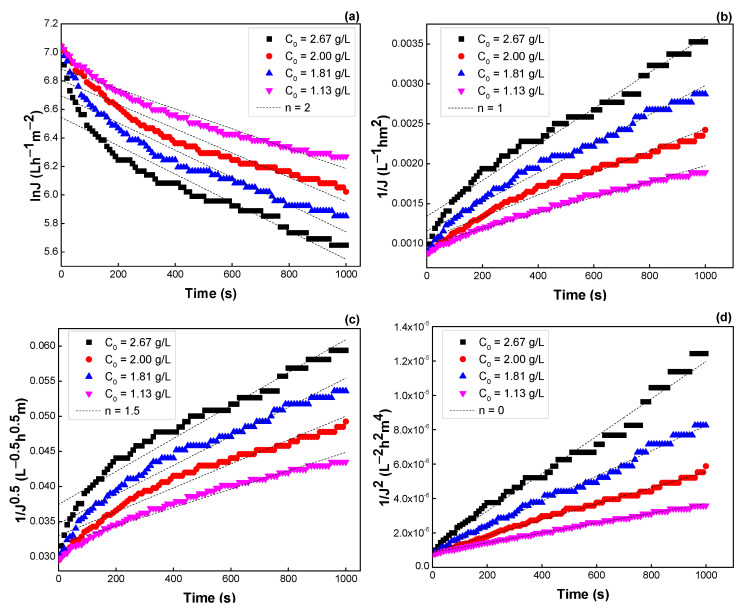
Effect of surfactin concentration on the flux predicted by the Hermia model: (**a**) *n* = 2, (**b**) *n* = 1, (**c**) *n* = 1.5, and (**d**) *n* = 0 (see Table 3).

The blocking mechanism was analyzed by varying initial surfactin concentration, TMP, and flow velocity. Table 4 and Figure 10 show the *R*^2^, *K* values, and the fitting of experimental data to different blocking mechanisms. The *R*^2^ values obtained using intermediate blocking (*n* = 1) and cake formation (*n* = 0) models are higher than those of using complete blocking, and standard blocking under the concentration ranges studied. That is, flux decline during cross-flow UF is ascribed to the deposit of large molecules on the membrane surface and gradually formed cake layer. Flux decline was more serious at higher surfactin concentrations with higher *K* values.

**Figure 11:**
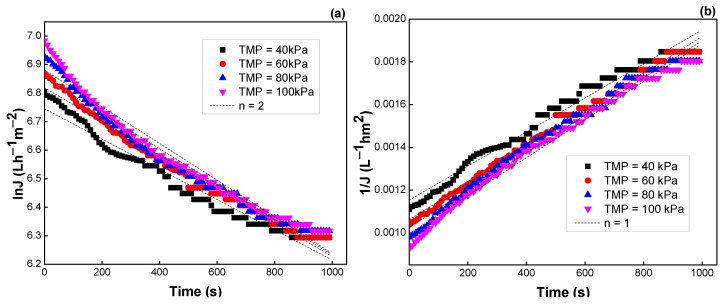

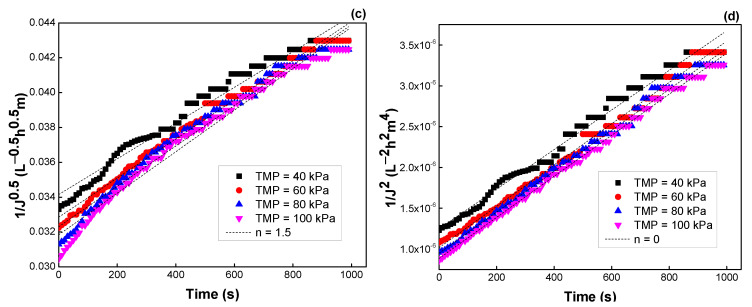
Effect of TMP on the flux predicted by the Hermia model: (**a**) *n* = 2, (**b**) *n* = 1, (**c**) *n* = 1.5, and (**d**) *n* = 0 (see Table 3).

**Figure 12:**
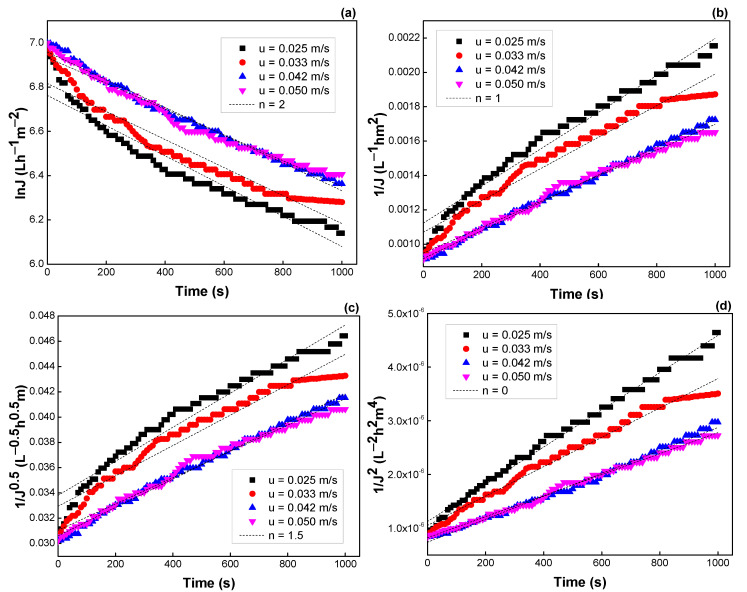
Effect of cross-flow velocity on the flux predicted by the Hermia model: (**a**) *n* = 2, (**b**) *n* = 1, (**c**) *n* = 1.5, and (**d**) *n* = 0 (see Table 3).

Similar results were observed when TMP and flow rate were varied. The *R*^2^ values, *K* values, and the fitting results are depicted in Figure 11 and Figure 12, Table 5 and Table 6, respectively. Both models of intermediate blocking (*n* = 1) and cake formation (*n* = 0) are also fitted better for flux prediction. Moreover, the *K* values were not changed much with TMP and velocity, indicating that flux decline was almost independent of these operating parameters.

From the results measured in this work and predicted by the Hermia model, flux decline in one-stage UF processes is initially caused by the resistance of intermediate blocking (*n* = 1) and then by that of cake formation (*n* = 0). The understanding of dominant fouling mechanisms by the Hermia model helps us to calculate the fluxes that can close to the measured values. Table 7 presents the suggested period that intermediate blocking and cake formation dominate. Apparently, the time for such a transition changes with the feed surfactin concentration.

### 3.4. Membrane Cleaning in Cross-Flow UF

Although the cross-flow UF process can reduce the deposit of the cake layer, it is still important for flux decline due to membrane fouling. Several common methods have been used to reduce fouling, including change in the interaction between particle surface, change in the hydrophilicity of the membrane, change in hydrodynamics in the module, and periodic cleaning [32,33]. The cleaning protocols suggested by membrane manufacturers include a series of acid-alkaline-flushing cycles depending on feed composition and membrane properties. According to the present results, an increase in *u* and TMP cannot efficiently increase the flux. Therefore, in situ periodic cleaning is needed to regain and maintain the flux.

**Figure 13:**
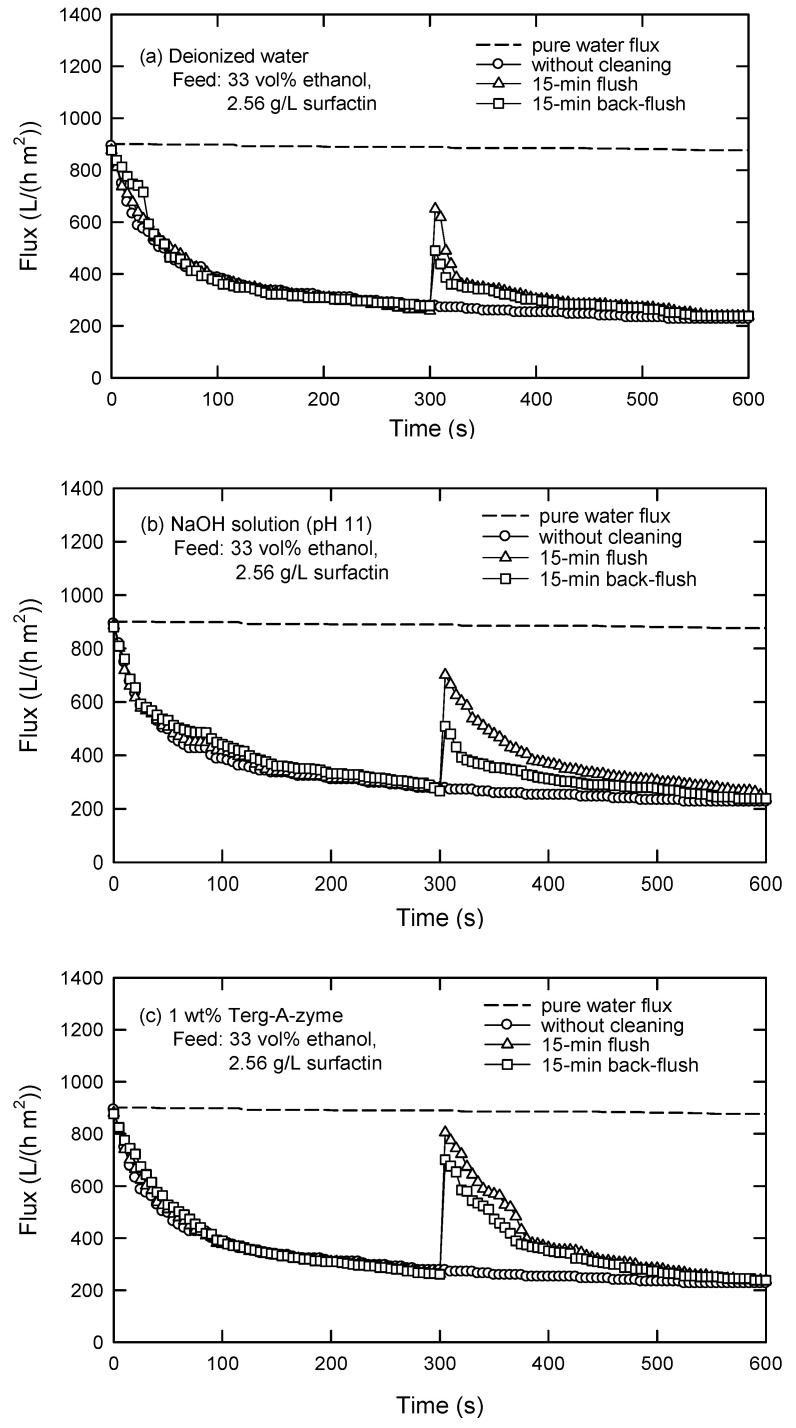
Cleaning of PES 100 membrane in cross-flow UF using (**a**) deionized water, (**b**) NaOH solution (pH 11), and (**c**) 1 wt.% Terg-A-zyme solution (TMP = 40 kPa, *u* = 0.033 m/s).

Three cleaning solutions were selected based on the characteristics of the precipitation-pretreated liquors; that is, 1 wt.% Terg-A-zyme (an enzyme solution), NaOH solution at pH 11, and deionized water. These solutions have been used in the UF of protein solutions for this purpose previously [18,34,35,36]. The effects of flush and back-flush on the flux at *C*_0_ = 2.5 g/L are illustrated in Figure 13. After 15-min flush and back-flush using deionized water, 75% and 59% of pure water fluxes can be recovered. Similarly, 84% and 78% of pure water fluxes are regained by flush and back-flush, respectively, with NaOH solution (pH 11), whereas 92% and 83% of pure water fluxes are recovered by using the enzymatic solution. In a word, the efficiency of membrane cleaning by flush is higher than that by back-flush. This is in agreement with the fouling mechanism, where large molecules are accumulated on the membrane surface instead of penetrating into the membrane pores. Apparently, the cleaning efficiency using enzyme solution is the best (Terg-A-zyme > NaOH > water), by either flush or back-flush.

As reported by Regula et al. [37], the cleaning mechanism while using NaOH is that the deposited species will be loosely bound and solubilized in NaOH solution. In the case of applying an enzyme solution, the mechanism is different. This agent acts as a catalyst in hydrolysis reaction, which promotes degradation of the fouled organic matter. The macromolecule blocks could be degraded and lifted off the membrane surface. The increased cleaning efficiency using Terg-A-zyme, in comparison with NaOH, and performance by flush rather than by back-flush indicate that the fouling was prominent because of the deposit of macromolecules on the membrane surface.

In a word, factors that are generally considered in membrane cleaning steps are cleaning agents and cleaning methods. Cleaning agents are chosen based on the feed composition and the properties of cleaning agents, including their dissolvability or reactivity with the foulants. Meanwhile, the cleaning methods (flush or back-flush) are adopted mainly based on the fouling mechanisms. Therefore, the predominant fouling mechanisms should be understood beforehand. Anyway, NaOH solution (pH 11) is the most suitable candidate when cost reduction and surfactin recovery are simultaneously considered.

## 4. Conclusions

A target feed containing surfactin was obtained by dissolving the precipitate in NaOH solution after the culture liquor of *Bacillus subtilis* ATCC 21332 was centrifuged and precipitated by acid. The one-stage UF process was then used as a tool for recovering surfactin from such treated liquor. In the surfactin concentration less than 0.75 g/L, dead-end UF tests simply showed that primary recovery of surfactin in the permeate was acceptable (>70%) when PES membrane with 100-kDa MWCO (PES 100) was used and 33 vol% ethanol was added in the feed. Fouling analysis also indicated that flux decline with all used membranes, including PES 100, was dominantly caused by cake formation. Under the conditions studied of surfactin concentration, applied pressure, and flow velocity, the Hermia model further confirmed that flux decline in the present one-stage UF processes was initially ascribed to the resistance of intermediate blocking and then to that of cake formation.

Cross-flow UF tests further confirmed the application potential and highlighted more advantages. Owing to the differences in operating dynamics of the dead-end and cross-flow modes, significant differences in both solute rejection and permeate flux were observed. Particularly, 75% of surfactin was recovered by cross-flow UF, much higher than that by dead-end UF (55%) at corresponding feed concentrations. Moreover, the steady flux obtained in cross-flow UF was almost ten times higher than that that in dead-end UF. An in situ membrane flush with NaOH solution (pH 11) was finally recommended, which could recover 84% of the pure water flux.

## Figures and Tables

**Table 1:** Components and their molecular weights (the numeral in the parentheses, in g/mol) in the raw *B. subtilis* ATCC 21332 fermentation broth [19].

Macromolecules	Mid-molecules	Small molecules
surfactin micelles (30,000–100,000)	surfactin monomers (1036)	MS medium (80–400)
polysaccharides		alcohols (46)
proteins		glycine (75)
peptides		alanine (89)
		phosphate (100)
		serine (105)
		threonine (119)
		phthalic acid (150)
		amino acid (200)

**Table 2:** MFI values determined using various UF membranes.

Feed Surfactin, *C*_0_	UF Membrane	MFI (h/m^6^)	Correlation Coefficient
0.25 g/L	PES 100	19.8	0.9998
	PAN 100	21.0	0.9995
	PVDF 30	32.9	0.9991
1.0 g/L	PES 100	9.1	0.9991
	PES 50	11.2	0.9971
	YM 30	20.9	0.9970

**Table 3:** Linear forms for different blocking mechanisms according to the Hermia model.

Model	*n* Value	Linear Fitting Equation
Complete blocking	*n* = 2	$\ln J=\ln J_{0}+K_{c}t$
Intermediate blocking	*n* = 1	$\left(1/J\right)=\left(1/J_{0}\right)+K_{i}t$
Standard blocking	*n* = 1.5	$\left(1/\sqrt{J}\right)=\left(1/\sqrt{J_{0}}\right)+K_{s}t$
Cake formation	*n* = 0	$\left(1/J^{2}\right)=\left(1/J_{0}^{2}\right)+K_{cf}t$

**Table 4:** *R*^2^ values and *K* constants in the Hermia model at different surfactin concentrations.

*C*_0_ (g/L)	Term	*n* = 2	*n* = 1	*n* = 1.5	*n* = 0
2.67	*R* ^2^	0.8981	0.9731	0.9454	0.9799
	*K*	9.96 × 10^−4^	2.25 × 10^−6^	2.34 × 10^−5^	1.09 × 10^−8^
2.00	*R* ^2^	0.9174	0.9718	0.9487	0.9941
	*K*	8.50 × 10^−4^	1.38 × 10^−6^	1.70 × 10^−5^	4.67 × 10^−9^
1.81	*R* ^2^	0.9167	0.9786	0.9542	0.9926
	*K*	9.55 × 10^−4^	1.82 × 10^−6^	2.07 × 10^−5^	7.33 × 10^−9^
1.13	*R* ^2^	0.9455	0.9807	0.9657	0.9964
	*K*	6.98 × 10^−4^	9.85 × 10^−7^	1.31 × 10^−5^	2.86 × 10^−9^

**Table 5:** *R*^2^ values and *K* constants in the Hermia model at different TMPs.

TMP (kPa)	Term	*n* = 2	*n* = 1	*n* = 1.5	*n* = 0
40	*R^2^*	0.9614	0.9789	0.9712	0.9880
	*K*	−5.30 × 10^−4^	7.96 × 10^−7^	1.02 × 10^−5^	2.23 × 10^−9^
60	*R^2^*	0.9817	0.9939	0.9893	0.9944
	*K*	−5.91 × 10^−4^	8.57 × 10^−7^	1.12 × 10^−5^	2.53 × 10^−9^
80	*R^2^*	0.9662	0.9868	0.9782	0.9941
	*K*	−6.19 × 10^−4^	8.73 × 10^−7^	1.16 × 10^−5^	2.51 × 10^−9^
100	*R^2^*	0.9646	0.9885	0.9785	0.9970
	*K*	−6.43 × 10^−4^	8.90 × 10^−7^	1.19 × 10^−5^	2.52 × 10^−9^

**Table 6:** *R*^2^ values and *K* constants in the Hermia model at different cross-flow velocities.

Flow Velocity (m/s)	Term	*n* = 2	*n* = 1	*n* = 1.5	*n* = 0
0.025	*R^2^*	0.9294	0.9728	0.9541	0.9933
	*K*	−6.83 × 10^−4^	1.07 × 10^−6^	1.35 × 10^−5^	3.47 × 10^−9^
0.033	*R^2^*	0.9294	0.9652	0.9493	0.9855
	*K*	−6.33 × 10^−4^	9.20 × 10^−7^	1.20 × 10^−5^	2.74 × 10^−9^
0.042	*R^2^*	0.9876	0.9977	0.9945	0.9931
	*K*	−6.27 × 10^−4^	8.10 × 10^−7^	1.12 × 10^−5^	2.14 × 10^−9^
0.050	*R^2^*	0.9736	0.9899	0.9831	0.9955
	*K*	−5.82 × 10^−4^	7.49 × 10^−7^	1.04 × 10^−5^	1.96 × 10^−9^

**Table 7:** The transition of fouling mechanisms with time at different feed concentrations.

*C*_0_ (g/L)	Intermediate Modeling (Time Range, s)	*R*^2^ (-)	Cake Modeling (Time Range, s)	*R*^2^ (-)
2.67	0–30	0.9985	30–1000	0.9781
2.00	0–200	0.9892	200–1000	0.9915
1.81	0–130	0.9835	130–1000	0.9891
1.13	1–300	0.9891	300–1000	0.9928

## Data Availability

The data presented in this study are available on request from the corresponding author.
